# An implementation algorithm to improve skin‐to‐skin practice in the first hour after birth

**DOI:** 10.1111/mcn.12571

**Published:** 2017-12-12

**Authors:** Kajsa Brimdyr, Karin Cadwell, Jeni Stevens, Yuki Takahashi

**Affiliations:** ^1^ Healthy Children Project East Sandwich Massachusetts USA; ^2^ School of Nursing and Midwifery University of Western Sydney Sydney New South Wales Australia; ^3^ Nagoya University Graduate School of Medicine Nagoya Japan

**Keywords:** algorithm, birth, caesarean, skin‐to‐skin, vaginal

## Abstract

Evidence supporting the practice of skin‐to‐skin contact and breastfeeding soon after birth points to physiologic, social, and psychological benefits for both mother and baby. The 2009 revision of Step 4 of the WHO/UNICEF “Ten Steps to Successful Breastfeeding” elaborated on the practice of skin‐to‐skin contact between the mother and her newly born baby indicating that the practice should be “immediate” and “without separation” unless documented medically justifiable reasons for delayed contact or interruption exist. While in immediate, continuous, uninterrupted skin‐to‐skin contact with mother in the first hour after birth, babies progress through 9 instinctive, complex, distinct, and observable stages including self‐attachment and suckling. However, the most recent Cochrane review of early skin‐to‐skin contact cites inconsistencies in the practice; the authors found “inadequate evidence with respect to details … such as timing of initiation and dose.” This paper introduces a novel algorithm to analyse the practice of skin to skin in the first hour using two data sets and suggests opportunities for practice improvement. The algorithm considers the mother's Robson criteria, skin‐to‐skin experience, and Widström's 9 Stages. Using data from vaginal births in Japan and caesarean births in Australia, the algorithm utilizes data in a new way to highlight challenges to best practice. The use of a tool to analyse the implementation of skin‐to‐skin care in the first hour after birth illuminates the successes, barriers, and opportunities for improvement to achieving the standard of care for babies. Future application should involve more diverse facilities and Robson's classifications.

## 1 INTRODUCTION

The WHO/UNICEF Baby‐Friendly Hospital Initiative integrates the “Ten Steps to Successful Breastfeeding” into practice. A 2009 revision offered specified guidance for Step 4, practices in the first hour after birth: place all babies “in skin‐to‐skin contact … immediately or within five minutes after birth” and continue “without separation for an hour or more, unless there were medically justifiable reasons” (World Health Organization & UNICEF, 2009, p. 34). The Baby‐Friendly USA (2016) Step 4 standard specifies *immediate*, *continuous*, and *uninterrupted* skin‐to‐skin contact (SSC) between the mother and her newly born infant after birth “unless there are documented medically justifiable reasons for delayed contact or interruption.” The Australian Baby‐Friendly Health Initiative recommends “immediate skin‐to‐skin contact on the mother's chest after birth and that the baby is allowed to remain there without interruption or separation” unless “a medically initiated procedure is required” (Australian College of Midwives, n.d., p. 22). The Japanese Baby‐Friendly Hospital Initiative guidelines follow the WHO 2009 revision and include the suggestion of a delay in routine care unless there is a medical reason for separation (Igaku‐shoin, 2009; Japan Society of Perinatal and Neonatal Medicine et al., 2012).

Echoes of Step 4 guidance are found in the policy documents and statements of professional organizations. For example, The Academy of Breastfeeding Medicine Model Policy states “At birth or soon thereafter all newborns, if baby and mother are stable, will be placed skin‐to‐skin with the mother … allow uninterrupted mother–infant contact and breastfeeding …” (ABM Protocol Committee, 2010, p. 173). The American Academy of Pediatrics Section on Breastfeeding Model Policy concurs, “Healthy term newborns with no evidence of respiratory compromise will be placed and remain in direct skin‐to‐skin contact with their mothers immediately after delivery until the first feeding is accomplished, unless medically contraindicated” (QuIIN [Quality Improvement Innovation Network] & American Academy of Pediatrics, 2009, p. 1). An official practice brief from the Association of Women's Health, Obstetric and Neonatal Nurses recommends immediate SSC that is uninterrupted and continues for the “first hour of life or until the first breastfeeding is completed” (AWHONN, 2015). The Academy of Obstetricians and Gynecologists in the Committee Opinion titled “Optimizing Support for Breastfeeding as Part of Obstetric Practice” suggests that SSC care should begin “early” (American Congress of Obstetricians & Gynecologists, 2016). The International Childbirth Education Association Position Paper on SSC directs that it begin “immediately after the birth of the baby” (Lowrie, 2015, p. 1), and the Lamaze Healthy Birth Practice #6 warns against interruption: “Disrupting or delaying skin‐to‐skin care may suppress a newborn's innate protective behaviors, lead to behavioral disorganization, and make self‐attachment and breastfeeding more difficult” (Lamaze International, n.d., p. 212). A collaborative document “Points to Bear in Mind in Regard to the Implementation of ‘Early Mother–Infant Skin‐to‐Skin Contact’” was published by the major Japanese professional organizations involved with birth and contains implementation strategies that include starting as soon as possible after birth and continuing until breastfeeding is complete (Japan Society of Perinatal and Neonatal Medicine et al., 2012).

While in immediate, continuous uninterrupted SSC with mother in the first hour after birth, babies progress through nine instinctive, complex, distinct, and observable stages (Table 1) that have been documented elsewhere (Widström et al., 2011). In Stage 7, the baby finds the nipple and licks, mouths, massages, and becomes familiar with the mother's breast; in Stage 8, the infant self‐attaches and suckles. Uncompromised term newborns go through these stages at varying rates and usually achieve suckling within 60 to 90 min after birth (Widström et al., 2011; Widström et al., 1987). Interruption, such as non‐emergent newborn care, is a modifiable practice that has been linked to decreased achievement of suckling (Robiquet et al., 2016). Breastfeeding within the first hour has been shown to have an inverse relationship with breastfeeding difficulties (Bramson et al., 2010) and neonatal mortality (Edmond et al., 2006; Wallace, Crear‐Perry, Richardson, Tarver, & Theall, 2017).

**Table 1:** Widström's 9 Stages

Babies progress through nine observable, instinctive stages during the first hour after birth when in immediate, continuous, and uninterrupted skin‐to‐skin contact with the mother. Stage 8 is suckling, the first experience of breastfeeding.
1. The birth cry is a distinct and specific cry as the baby's lungs expand for the first time.
2. Relaxation is a time immediately after the birth cry ends, when the baby becomes still and has no visible movements.
3. Awakening begins as the baby opens the eyes for the first time, blinks, has small mouth movements, and limited hand and shoulder motions.
4. Activity involves larger body movements, including whole arm motions, specific finger movements, shoulder motion, head lifting, and stable open eyes.
5. Rest could happen at any point during the first hour, interspersed between stages or as a transition between stages.
6. Crawling involves the baby moving purposely towards the breast and nipple. It could be accomplished through sliding, leaping, bobbing, or pushing.
7. Familiarization is a stage at the mother's nipple where the baby licks, tastes, touches, and moves around the nipple and areola area.
8. Suckling involves the baby self‐attaching to the nipple and initiating breastfeeding.
9. Sleeping is an involuntary activity of the baby around 1.5 to 2 hr after birth.

*Note*. Adapted from Widström, A. ‐M., Lilja, G., Aaltomaa‐Michalias, P., Dahllöf, A., Lintula, M., and Nissen, E. Newborn behaviour to locate the breast when skin‐to‐skin: A possible method for enabling early self‐regulation. Acta Paediatr Oslo Nor 1992. 2011 Jan, 100(1): 79–85.

Evidence supporting the biologically normal (Bergman, Linley, & Fawcus, 2004) practice of SSC after birth points to benefits for both mother and baby; SSC holding decreases the baby's stress of being born (Bystrova et al., 2003; Takahashi, Tamakoshi, Matsushima, & Kawabe, 2011) and promotes more optimal thermoregulation (Beiranvand, Valizadeh, Hosseinabadi, & Pournia, 2014) continuing even through the first days (Nimbalkar et al., 2014). Babies cry less (Christensson, Cabrera, Christensson, Uvnäs‐Moberg, & Winberg, 1995; Mazurek et al., 1999) and have improved the cardiopulmonary dynamics during the early hours after birth (Takahashi et al., 2011). SSC has been shown to increase breastfeeding initiation and exclusive breastfeeding, reduce formula supplementation in hospital, lead to an earlier successful first breastfeed (Bramson et al., 2010; Crenshaw et al., 2012; Mahmood, Jamal, & Khan, 2011; Marín Gabriel et al., 2010; Mikiel‐Kostyra, Mazur, & Bołtruszko, 2002; Srivastava, Gupta, Bhatnagar, & Dutta, 2014), and promote more optimal suckling (Righard & Alade, 1990). For the mother, early SSC leads to earlier expulsion of the placenta (Marín Gabriel et al., 2010), reduced bleeding (Dordević, Jovanović, & Dordević, 2008), lowered maternal stress levels (Handlin et al., 2009), and enhanced breastfeeding self‐efficacy (Aghdas, Talat, & Sepideh, 2014). The contact of the baby while skin to skin with the mother induces a rise in the hormone oxytocin, which in turn leads to more social responsiveness and may also promote parenting behaviours (Uvnäs‐Moberg, 1998; Winberg, 2005), bonding, and attachment (Affonso, Wahlberg, & Persson, 1989).

In spite of the supportive policies, professional statements, and the numerous advantages of SSC described in the literature, the most recent Cochrane review of early SSC for mothers and their healthy newborn infants (Moore, Bergman, Anderson, & Medley, 2016) cites inconsistencies in the practice. For example, only 47% of the 38 included trials selected for the systematic review reported that SSC began “early” or “immediately,” meaning that after the birth, the baby had been carefully dried and placed, without delay, on the mother's abdomen (in the case of a vaginal birth) or on the mother's chest above the drape (in the case of a caesarean birth). In addition, 66 studies were assessed and excluded from the Cochrane review. The primary reason for exclusion was that “ … the investigators did not state that the infants in the intervention group received immediate or early skin‐to‐skin contact” (Moore et al., 2016). In some published accounts of SSC, the duration has ranged from as few as 15 min (De Chateau & Wiberg, 1977; Vaidya, Sharma, & Dhungel, 2005) to a mean of more than 30 hr, Syfrett 1993 as cited in Moore et al. (2016). The Cochrane review concludes:

Despite our concerns about the quality of the studies, and since we found no evidence of harm in any included studies, we conclude the evidence supports that early SSC should be normal practice for healthy newborns including those born by cesarean and babies born early at 35 weeks or more. (Moore et al., 2016, p. 3)

However, the authors found “inadequate evidence with respect to details … such as timing of initiation and dose” relative to outcomes (Moore et al., 2016, p. 29).

Although giving birth via caesarean is a well‐documented barrier to SSC in the first hour (Stevens, Schmied, Burns, & Dahlen, 2014), it is not known whether other obstetrical conditions affect the practice. Robson's criteria have been used most often prebirth to prospectively “compare CS rates in a consistent and action‐oriented manner” (Betrán, Vindevoghel, Souza, Gülmezoglu, & Torloni, 2014, p. 1). The Robson 10‐group classification system utilizes straightforward obstetric parameters such as parity, singleton or multiple pregnancies, gestational age, spontaneous or induced labour, prior caesarean section, breech fetus, and abnormal positioning, including transverse or oblique. The use of the Robson classification system decreases interpretation and allows comparison across hospital systems, states, and countries. The modified Robson criteria for Canada (Farine & Shepherd, 2012) combine the original 2001 10‐group classification with suggestions proposed in subsequent years allowing for subcategories to be separated and compared (Table 2). Tracking the Robson criteria can be used to determine if different groups receive a different experience during the first hour after birth.

**Table 2:** The Canadian modified Robson criteria (adapted from Farine & Shepherd, 2012)

Group	Description
1	Nullipara, singleton cephalic, ≥37 weeks, spontaneous labour
2	Nullipara, singleton cephalic, ≥37 weeks A. Induced B. Caesarean section before labour
3	Multipara, singleton cephalic, ≥37 weeks, spontaneous labour
4	Multipara, singleton cephalic, ≥37 weeks A. Induced B. Caesarean section before labour
5	Previous caesarean section, singleton cephalic, ≥37 weeks A. Spontaneous labour B. Induced C. Caesarean section before labour
6	All nulliparous breeches A. Spontaneous labour B. Induced C. Caesarean section before labour
7	All multipara breeches (including previous caesarean section) A. Spontaneous labour B. Induced C. Caesarean section before labour
8	All multiple pregnancies (including previous caesarean section) A. Spontaneous labour B. Induced C. Caesarean section before labour
9	All abnormal lies (including previous caesarean section but excluding breech) A. Spontaneous labour B. Induced C. Caesarean section before labour
10	All singleton cephalic, ≤36 weeks (including previous caesarean section) A. Spontaneous labour B. Induced C. Caesarean section before labour

**Figure 1:**
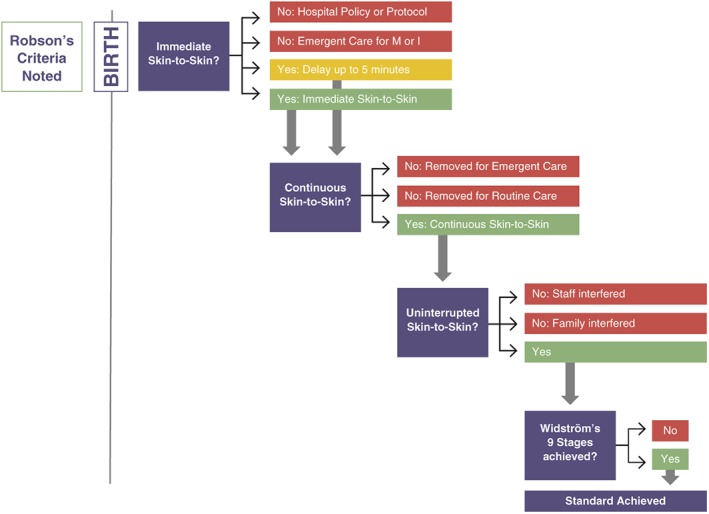
Healthy Children Project's Skin‐to‐Skin Implementation Algorithm—© Healthy Children Project, used with permission

In light of our experience researching and implementing SSC (Brimdyr et al., 2015; Brimdyr, Widström, Cadwell, Svensson, & Turner‐Maffei, 2012; Stevens, Schmied, Burns, & Dahlen, 2016; Takahashi et al., 2011) and in consideration of the findings of the Cochrane team (Moore et al., 2016), we present a novel algorithm, *Healthy Children Project's Skin‐to‐Skin Implementation Algorithm* (HCP‐S2S‐IA; Figure 1), which considers the mothers' condition as she begins the birthing experience according to Robson criteria (Table 2) and then, using the tool, plots the experience of each dyad in regard to immediate, continuous, and uninterrupted SSC after birth. We conducted iterative analyses of videotapes of immediate, uninterrupted, and continuous SSC in the first hour in two hospitals, one in Japan with mothers who gave birth vaginally and one in Australia with mothers who gave birth via caesarean.

**Key messages**

- Skin to skin contact and breastfeeding soon after birth points to physiologic, social, and psychological benefits for both mother and baby.
- The most recent Cochrane review of early skin to skin contact cites inconsistencies in the practice.
- A novel algorithm is able to analyze the practice of skin to skin in the first hour and suggest opportunities for practice improvement.
- The tool allows for the analysis of implementation of the practice of skin to skin care in the first hour and illuminate the successes, barriers, and opportunities for improvement in achieving the standard of care for babies.

## 2 METHODS

### 2.1 Data collection—Japan

For the purpose of examining the SSC implementation algorithm, we are analysing data from a Japanese study on newborn behaviour after vaginal birth from a Baby‐Friendly designated hospital in Nishio, Japan. A convenience sample of 14 clinically uncomplicated primipara and multipara mothers gave informed consent to participate into the study, which included videotaping infants during the first hour or so after birth while the babies were in SSC with their mother. The study's inclusion criteria included Japanese‐speaking women ≥18 years of age who were healthy and non‐smokers. Both primipara and multipara mothers were included if they planned a normal birth with no analgesia during labour. The infants were eligible if they were healthy and born at term and could continue in the study if they had an Apgar score of at least eight at 1 min after birth. Each dyad received a unique code within the study that was also associated with the video record of the first hour after birth. They were placed in SSC, as per hospital routine. The study did not change any hospital protocols or routines, with the exception of the addition of the video recording of the baby for the first hour after birth while in SSC with the mother.

Immediately after the birth, the newborn was placed in SSC ventrally on the mother's abdomen, dried, and covered with a warm blanket. Hospital protocol stated that the baby would remain in SSC continuously with the semireclined mother for at least the first hour after birth unless there was a medical reason to interrupt. The baby was monitored using Pulse Oximetry (Covidien‐Nellcor and Puritan Bennett, Boulder, USA) following the Japanese guideline for early SSC (Wyllie et al., 2015). The baby was allowed to move, uninterrupted, through Widström's 9 Stages. The research protocol provided that if the baby was removed by the nurse or delivery ward staff for more than 60 min, the video recording would be stopped. The dyad would then be described as “removed for medical reasons.” Demographics and labour medications were collected from the Electronic Medical Record System. The study was approved by the Ethics Review Committee of the Nagoya University School of Medicine, Nagoya, Japan.

The infant's behaviour while in SSC with mother was video recorded for 1 hr. Subsequently, iterative analysis was performed by two research assistants who had been trained to identify each of Widström's 9 Stages of Newborn Behaviour. The training involved viewing a professional video (Brimdyr, Wiström, & Svensson, 2011) that defined and illustrated each stage and then attending a workshop about Widström's 9 Stages. The research assistants separately and independently coded all of the blinded video recordings for the nine stages using MAXQDA 11.0.2, 2013, a professional qualitative data analysis software. Although behaviours of staff, family, and the mother who interacted with the baby were noted, this paper reports only on the experience of the baby during the first hour with the expectation of the baby achieving all nine stages.

### 2.2 Data collection—Australia

We also analysed data from an Australian study conducted at a metropolitan public hospital in Sydney, Australia, that has approximately 3,700 births a year. The Baby‐Friendly Health Initiative in Australia recommends that for caesarean births, the “baby is placed skin‐to‐skin on the mother's chest whilst she is on the theatre table, immediately after or within 5 minutes” (Australian College of Midwives, n.d., p. 22). This study focused on SSC in the operating theatre and in recovery after elective repeat caesarean. Twenty‐one women who planned a repeat caesarean birth participated. Sixteen of the women gave written consent to field notes being collected and being videotaped, whereas the remaining five women gave written consent to only field notes being collected. Data collection was focused on maternal and support person contact with the baby for up to 2 hr immediately after the birth. The study's inclusion criteria included mothers who were planning an uncomplicated caesarean birth following a previous caesarean birth, who were between 18 and 40 years of age, had a singleton pregnancy, and planned to breastfeed. The specifics of the research have been reported elsewhere (Stevens et al., 2016). Standard midwifery care at this hospital included the initiation of SSC in the recovery area. This was facilitated by a post‐natal midwife after the birth unit midwife transferred care to them in recovery or by a caseload midwife (a continuity of care midwife) who was expected to stay with the woman in the operating theatre and on into the recovery area. Despite this being the standard midwifery care, it was known anecdotally that some midwives initiated SSC in the operating theatre. The study did not change any hospital protocols or routines, with the exception of collecting data, and for most mothers, videotaping of all maternal and support person contact with the baby in the first 2 hr. The methodology is detailed elsewhere (Stevens et al., 2014). The study was approved by the Human Research Ethics Committee from the hospital, Study No. 13/47‐HREC/13/ … /102 and Western Sydney University, Study No. H10482.

Analysis of the video and audio recordings was conducted through NVivo10 and analysed through critical ethnographic techniques. Furthermore, the timing of relevant events, including the timing of all maternal–infant contact, was recorded. For the purposes of this analysis, the video footage and field notes provided the data.

### 2.3 Method of analysis

Data regarding the experience of the mothers and babies from both the Japanese data set and the Australian study were analysed separately to elucidate the experience of the mothers and babies after birth. Then, using the HCP‐S2S‐IA algorithm, the dyads were plotted individually. The algorithm is colour coded—blue boxes and arrows point to the pathway of best practice. Red shows how and when the dyad has left the best practice trail. Yellow indicates that the dyad has encountered situations that are not best practice but may not be precluded from the achievement of immediate, continuous, and uninterrupted SSC in the first hour. Green boxes indicate best practice in each parameter. After the numbers have been entered, the algorithm is used to review the numbers and reflect on institutional barriers to best practice. The mothers were classified according to Robson's criteria as modified for Canada. The Robson's criteria were applied to each step in the algorithm. For ease of use, the Robson's criteria chart is also colour coded with red, yellow, and green, to match the steps of the algorithm. This allows for a second dimension of inquiry using the algorithm; is there a pattern of outcome based on Robson Criteria?

The first element, after the birth, is Immediate Skin‐to‐Skin. Here, we ask, was the newborn placed immediately (within 5 min) on the mother's chest? Did hospital protocol or policy preclude the pair from receiving immediate SSC at the time of the birth? These would be included as “No: Hospital Policy or Protocol” on the algorithm.

Newborns for whom hospital policy would have allowed SSC but who may not have received it are also considered in the immediate section of the algorithm This lack of SSC due to emergent care for the mother or infant results in the pair being removed from the best practice pathway if separation lasts for more than 5 min. They would then be categorized as “No: Emergent Care for Mother or Infant.” If the separation results in a delay of less than 5 min, the pair is categorized as yellow—a caution for the hospital to review—but the dyad continues on the blue best practice path.

The next element of the model considers continuous SSC during the full first hour or so. If the newborn is removed from SSC either for emergent or routine care any time within the first hour or so after birth, it is recorded.

The algorithm considers the standard of uninterrupted or undisturbed SSC next. Dyads are recategorized if staff interfere with the newborn during the first hour or if the mother or family interferes with the newborn's innate behaviours. Staff “helping” the newborn to latch would be considered interrupting, because the SSC standard includes newborn crawling, familiarizing, and self‐attaching.

Best practice is ultimately measured through the achievement of Widström's 9 Stages during the first hour or so after birth. Immediate, continuous, and uninterrupted practice optimizes the infant's ability to achieve this goal.

If a dyad experienced immediate, continuous, uninterrupted SSC for the first hour or so after birth and progressed through Widström's Stages and suckled, they have achieved the standard of best practice.

## 3 RESULTS

### 3.1 Results—Japanese data set

**Figure 2:**
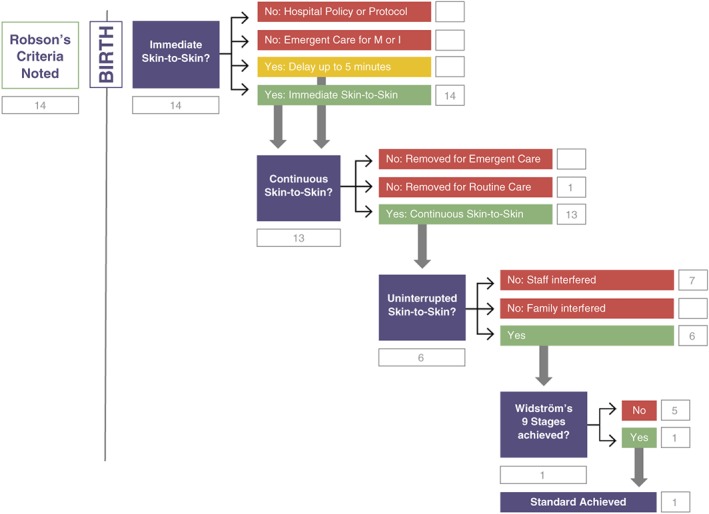
Healthy Children Project's Skin‐to‐Skin Implementation Algorithm with Japanese vaginal birth data

Analysis of the Japanese dyads is presented in Figure 2. Fourteen mothers consented to participate in the study. They are categorized in Table 3, column “Consenting, prebirth” according to Robson's criteria. All mothers had a vaginal birth. Five mothers are included in Group 1, nulliparas with spontaneous labour; eight are multiparas with a spontaneous labour, and one is a multipara who was induced.

**Table 3:** Categorizing the initial dyads and where they were recategorized across Healthy Children Project's Skin‐to‐Skin Implementation Algorithm: Japan

Group	Description	Prebirth	Immediate hospital policy at birth	Immediate emergent care immediately after birth	Immediate delayed <5 at birth	Immediate skin to skin	Continuous: Removed for emergent care	Continuous: Removed for routine care	Continuous	Uninterrupted: Staff interfered	Uninterrupted: Family interfered	Uninterrupted	Not achieved nine stages	Met standard
1	Nullipara, singleton cephalic, ≥37 weeks, spontaneous labour	5				5			5	2		3	3	
2	Nullipara, singleton cephalic, ≥37 weeks A. Induced B. Caesarean section before labour	0												
3	Multipara, singleton cephalic, ≥37 weeks, spontaneous labour	8				8		1	7	5		2	1	1
4	Multipara, singleton cephalic, ≥37 weeks A. Induced B. Caesarean section before labour	A. 1				A. 1			A. 1			1	A. 1	
5	Previous caesarean section, singleton cephalic, ≥37 weeks A. Spontaneous labour B. Induced C. Caesarean section before labour													
6	All nulliparous breeches A. Spontaneous labour B. Induced C. Caesarean section before labour													
7	All multipara breeches (including previous caesarean section) A. Spontaneous labour B. Induced C. Caesarean section before labour													
8	All multiple pregnancies (including previous caesarean section) A. Spontaneous labour B. Induced C. Caesarean section before labour													
9	All abnormal lies (including previous caesarean section but excluding breech) A. Spontaneous labour B. Induced C. Caesarean section before labour													
10	All singleton cephalic, ≤36 weeks (including previous caesarean section) A. Spontaneous labour B. Induced C. Caesarean section before labour													
Total mothers		14	0	0	0	14	0	1	13	7	0	6	5	1

All 14 babies were immediately placed in SSC with their mother. None were removed due to hospital policy or emergent care. Thirteen of the 14 babies received continuous SSC with their mother. One baby was removed at 50 min. Seven of the 13 remaining newborns were interrupted by staff who “helped” the newborn to breastfeed during the first hour. Six newborns were uninterrupted. One of the newborns who had immediate, continuous, uninterrupted SSC progressed through Widstrom's 9 Stages and achieved the standard of self‐attachment and suckling. The other five newborns did not progress past the Activity Stage (Stage 4).

### 3.2 Results—Australian study

**Figure 3:**
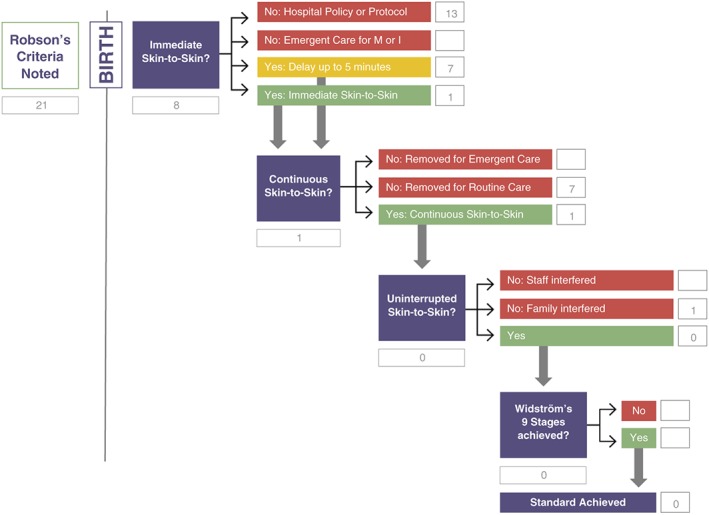
Healthy Children Project's Skin‐to‐Skin Implementation Algorithm with Australian caesarean birth data

Analysis of the Australian study is presented in Figure 3. Twenty‐one mothers consented to participate in the study. Table 4 categorizes the 21 mothers according to Robson's criteria. All mothers had a repeat, elective caesarean section and were consented before surgery (Robson's category 4B).

**Table 4:** Categorizing the initial dyads and where they were recategorized across Healthy Children Project's Skin‐to‐Skin Implementation Algorithm: Australia

Group	Description	Prebirth	Immediate hospital policy at birth	Immediate emergent care immediately after birth	Immediate delayed <5 at birth	Immediate skin to skin	Continuous: Removed for emergent care	Continuous: Removed for routine care	Continuous	Uninterrupted: Staff interfered	Uninterrupted: Family interfered	Uninterrupted	Not achieved nine stages	Met standard
1	Nullipara, singleton cephalic, ≥37 weeks, spontaneous labour													
2	Nullipara, singleton cephalic, ≥37 weeks A. Induced B. Caesarean section before labour													
3	Multipara, singleton cephalic, ≥37 weeks, spontaneous labour													
4	Multipara, singleton cephalic, ≥37 weeks A. Induced B. Caesarean section before labour	A. 0 B. 21	A. B. 13		A. B. 7	A. B. 1		A. B. 8						
5	Previous caesarean section, singleton cephalic, ≥37 weeks A. Spontaneous labour B. Induced C. Caesarean section before labour													
6	All nulliparous breeches A. Spontaneous labour B. Induced C. Caesarean section before labour													
7	All multipara breeches (including previous caesarean section) A. Spontaneous labour B. Induced C. Caesarean section before labour													
8	All multiple pregnancies (including previous caesarean section) A. Spontaneous labour B. Induced C. Caesarean section before labour													
9	All abnormal lies (including previous caesarean section but excluding breech) A. Spontaneous labour B. Induced C. Caesarean section before labour													
10	All singleton cephalic, ≤36 weeks (including previous caesarean section) Spontaneous labour Induced Caesarean section before labour													
Total mothers		21	13		7	1		8						

One baby was immediately placed in SSC with mother. Seven were placed in SSC with mother within 5 min of birth. Twelve received SSC later than 5 min after the birth (between 5.01 and 65.05 min), and one did not receive any SSC within the first 2 hr after birth. Seven of the mothers who received immediate SSC, or SSC within 5 min of birth, did not experience continuous SSC because they received less than 45 min of initial SSC with mother. The newborns were removed from the mother for routine care. One baby was not removed for routine care. One mother requested the baby to be removed, and it was recorded as “interrupted” on the algorithm. None of the babies met the standard for SSC—immediate, continuous, uninterrupted SSC for at least an hour, progressing through Widström's 9 Stages, self‐attaching to the breast and suckling.

## 4 DISCUSSION

### 4.1 Discussion—Japan

The algorithm allows us to examine SSC during the first hour after birth in relation to the international standard of immediate, continuous, uninterrupted SSC with the goal of progressing to self‐attached suckling followed by sleep. By using the HCP‐S2S‐IA, the hospital can review practice in order to better understand their barriers to achieving the standard of care and more clearly identify where to direct interventions aimed at improvement.

A review of the algorithm (Figure 1) representing the data from the Japanese study indicates that the hospital practice succeeded with immediate SSC for mothers who gave birth vaginally. All newborns in the study were placed in SSC with mother within the first minutes after birth. Short delays (less than 5 min) were observed when the mother needed to move into a position that was more conducive to SSC, for example, after a mother gave birth on her hands and knees (ID‐YJ11), she manoeuvred carefully, with the assistance of the midwife, to lay on her back, in a slight incline. This took 3:14 min.

Hospital practices were also successful at supporting the continuous aspect of SSC. Only one infant was removed and that was for routine care at 50 min. Hospital practices could be reviewed to determine whether staffing issues or staff education would help to ensure continuous SSC for at least 60 min, or until the newborn falls asleep (around 90 min after birth), for all mothers.

More than half of the mothers in the Japanese data set were subject to interruption by staff during the first hour after birth. Two of the newborns were moved by the staff. The other five were moved and then latched by the staff to the mother's nipple. We speculate that this may be a result of the historic understanding Step 4 of the BFHI, “Help mothers initiate breastfeeding within a half‐hour of birth.” This could easily be interpreted as assisting a mother to latch her baby within 30 min of the birth. In 2009, WHO clarified this step, emphasizing SSC during the first hour or so after birth, although the wording of the step itself was unchanged (World Health Organization & UNICEF, 2009, p. 34). The use of HCP‐S2S‐IA highlights the need for staff education and skills regarding the 2009 changes to the interpretation of Step 4 of the BFHI.

Only one of the newborns who received immediate, continuous, uninterrupted SSC in the Japanese data set progressed through Widstrom's 9 Stages, self‐attached, and achieved the standard of suckling within the first hour as a continuum of instinctive behaviour. If babies do not achieve suckling, the hospital might closely examine practices and review elements that could be interfering with a newborn's instinctive behaviour. For example, synthetic oxytocin as well as epidurals containing fentanyl can change a newborn's behaviour during the first hour, resulting in the newborn not progressing to suckling (Brimdyr et al., 2015). Robson's criteria applied to the Japanese data set mothers indicates that, of the five mothers in this category, three are primiparas who were not induced and did not have exposure to epidural, one is a multipara who was not induced and did not have exposure to epidural, and one was a multipara who was induced but did not have exposure to epidural. Yet none of the five newborns in this category progressed past the Activity Stage (Stage 4). What else could be inhibiting the newborn's innate behaviour? The algorithm highlighted an area of research that could be proving a barrier to the otherwise implemented advantages of immediate, continuous, uninterrupted SSC. For example, infants who had naso‐oropharyngeal suctioning at birth were six times less likely to suckle effectively during the first hour after birth (Cantrill, Creedy, Cooke, & Dykes, 2014). One speculation is that the use of iodine during the birthing process, which has a strong and distinct odour, may be interfering with the instinctive behaviour of the newborn to smell the amniotic fluid and the colostrum/Montgomery gland scents as a directive to the breast (Porter, 2004; Porter & Winberg, 1999). Iodine has an intensifying impact on olfactory cells as well as a negative effect on eyesight. More exploration is needed to clarify the findings.

### 4.2 Discussion—Australia

We examined the data of Australian mothers who gave birth via elective caesarean in relation to the standard of immediate, continuous, uninterrupted SSC with the goal of progressing to self‐attached suckling followed by sleep. Only one newborn in the study was placed in SSC with a mother immediately after birth (Figure 3). Short delays (less than 5 min) were observed in seven of the mothers before they received their newborn in SSC in the operating theatre. Hospital practices prevented 13 of the dyads from receiving immediate SSC.

A further review of the HCP‐S2S‐IA indicates that hospital practices also interfered with the continuous aspect of SSC. Seven of the eight newborns who were placed in SSC within 5 min of birth were removed within the first hour. In this example, hospital practices might be reviewed to examine whether or not staffing or staff education would help to ensure continuous SSC for at least 60 min, or until the newborn breastfeeds and falls asleep (around 90 min after birth).

Examination of uninterrupted SSC in the Australian data set indicates that the single mother who did not have her newborn removed for routine care by the staff asked for the newborn to be removed due to nausea. According to analysis, 20 of the 21 Australian newborns were unable to complete at least 60 min of SSC due to hospital policies or routine care. The tool provides feedback to the hospital regarding the current barriers in implementing immediate, continuous, uninterrupted SSC after elective caesarean birth.

### 4.3 Discussion

#### 4.3.1 What the data as a whole says about the patterns of behaviour in this field

The algorithm allows review and deeper understanding of the barriers to best practice of immediate, continuous SSC during the first hour after birth. The first question concerns immediate SSC between the mother and baby. Was the newborn placed immediately (within 5 min) on the mother's chest? What could be preventing this implementation of best practice? This could be the case if the hospital has a protocol, for example, that includes babies born by caesarean in the classification of those who should not be eligible for immediate SSC. This was the case for a number of mothers in the Australian study. Other challenges could be a policy about births that occur in the Emergency Department or if certain anaesthesia was used. Perhaps the hospital has no policy about SSC as best practice, and implementation is based on staff preference or mother request. Does the facility not allow for staffing the required number of nurses to enable SSC? Perhaps swaddling or washing or routinely assessing every baby while on a warming table was required by protocol, or conducted by staff choice, before SSC could be started. These would be included as No: Hospital Policy or Protocol on the algorithm. This categorization could highlight for the hospital the barriers to best practice.

None of the separation in our two example cases revealed prevention of immediate SSC due to emergent care for the mother or infant. This would have been the categorization if newborn did not have an initial birth cry or had a low 1‐min Apgar requiring transfer to the NICU team, rather than to the mother's chest, or, the mother has haemorrhaged, requiring the newborn to be placed elsewhere. It is vital for a hospital to recognize if the lack of immediate SSC contact is due to hospital policy or a need for emergent care. Each reflects opportunities for more in depth review or quality improvement projects.

The next element of the model considers continuous SSC during the full first hour or so. Continuous SSC is vital for the warmth, respirations, and colonization of the newborn. Because the newborn will need to begin again the progression through Widström's 9 Stages after separation from the mother, any separation can be problematic because the newborn has limited time before falling asleep after birth. It is important to understand why the separation is occurring. Are there concerns about the infant's respirations? Are there concerns about the mother haemorrhaging? These would be examples of Emergent Care. None of the infants in these two examples were removed during the first hour for emergent care. If they had been, it would highlight a concern about the status of the mother and/or infant immediately after birth. Were the newborns removed for routine care? This occurred both in the Japanese data set and the Australian data set. Routine care during the first hour after birth can and should be conducted while mother and infant remain in SSC. This pathway of the algorithm highlights an opportunity for staff education.

The algorithm considers the standard of uninterrupted or undisturbed SSC next. Dyads are recategorized if staff interfere with the newborn during the first hour or if the mother or family interferes with the newborn's innate behaviours. This could be subtle or significant. It is possible for staff to shift a newborn, to take vital signs, for mother to eat and shift position all without disturbing the newborn through the progression of the nine stages. It is possible to move the mother from an operating table to the stretcher without separating or interfering with newborn behaviour. On the other hand, it is possible to disrupt a newborn when checking vitals or helping a mother to shift position. Staff gently helping to reposition a newborn's head to maintain a clear airway could be done in a way that does not interrupt the newborn. Flipping a baby over and rearranging the newborn's body could be very disruptive. Staff “helping” the newborn to latch would be considered interrupting, because the SSC standard includes newborn crawling, familiarizing, and self‐attaching. It is this “help” that is highlighted in the Japanese data, presenting an opportunity for staff education on the latest understanding of best practice.

The ultimate goal of immediate, continuous, uninterrupted SSC during the first hour after birth is manifested through the instinctive behaviour of the newborn during this time. If needed, this can be observed closely as Widström's 9 Stages. However, even when unobserved, this is the neurological pattern of behaviour that infants progress through during the first hour. Video allows us to examine this closely, but a secure knowledge of the instinctive behaviour allows us to understand when a newborn has, or has not, achieved all of the stages. Each stage is clear, even without close observation. Did the newborn open their eyes? Did the newborn move their arms and shift their body? Did the newborn progress to the breast? Did the newborn lick the nipple? Mothers, partners, and health care providers can know and understand whether the newborn progressed through the stages and achieved suckling and sleeping, without needing to stand over the dyad for the full hour. If a newborn has not achieved these stages—why would they not open their eyes? Why would they not crawl? Why would they not lick? Why would they not suckle?—needs to lead to further examination of these newborns, perhaps by examining their exposure to labour medications (Brimdyr et al., 2015), or the infant's physical condition, can give insight into their experience. A review of the Australian data shows that newborns never had the opportunity to consider the achievement of the standard, due to barriers during the first hour. Continued use of the tool would allow deeper understanding of the challenges, an opportunity to celebrate further progress, and a clear understanding of the goal of best practice. A review of the Japanese data highlights a challenge at the end of the algorithm. Why would newborns not demonstrate instinctive behaviour? Further exploration can focus on this question, to increase the benefits of the work of implementing SSC.

Although giving birth via caesarean is a well‐documented barrier to SSC in the first hour (Stevens et al., 2014), it is not known whether other obstetrical conditions affect the practice. The additional layer of Robson's criteria helps to highlight progress made in overcoming the known challenges of SSC after caesarean, as well as deepening the understanding of appropriate implementation of best practice with SSC to all mothers.

Research shows that dyads who experience immediate, continuous, uninterrupted SSC for the first hour or so after birth, and progress through Widström's Stages and suckle, achieve the standard of best practice. Why is there only one dyad who achieved this standard? Research studies highlight the inconsistencies of implementation of SSC, as highlighted in the most recent Cochrane review of early SSC for mothers and their healthy newborn infants (Moore et al., 2016), including the timing of initial SSC, the duration, and the concept of continuous contact. A study of Australian midwives highlighted their understanding of the importance of SSC, but not of the importance of “continuous, uninterrupted” SSC (Cantrill, Creedy, & Cooke, 2004). By highlighting the challenges of immediate, continuous, uninterrupted SSC, hospitals can illuminate where they are not yet meeting best practice, as well as understand a path forward towards best practice.

Although based on evidence, this algorithm is illustrated on only two sets of data. Implementation of the algorithm in different locations will strengthen the ability to compare and contrast strengths and barriers of different settings, allowing shared understanding of the challenges of implementing immediate, continuous, uninterrupted SSC. It is vital that the algorithm continues to focus on clinical practice, rather than the “result” of simply suckling, because that misunderstanding of the early interpretations of Step 4 of the BFHI resulted in helping the newborn to breastfeed, with unfortunate consequences.

## 5 CONCLUSION

We have developed and provided examples of use for a tool intended to improve implementation of the evidence‐based practice of SSC in the first hour after birth. The novel algorithm (HCP‐S2S‐IA) combines Robson's criteria for obstetric classification, parameters for best practice of SSC (immediate, continuous, and uninterrupted) along with Widström's 9 Stages in order to evaluate the experience of mothers and babies in the first hour after birth. Although the exemplar sample sizes were small, differences could be discerned, and opportunities for practice improvement can be elucidated. Larger samples with more varied Robson classifications are likely to assist hospital staff further in understanding the barriers within more discrete populations of mothers. Because every birthing facility has unique strengths and challenges, use of the algorithm periodically will allow new barriers to be documented and progress celebrated. This simple algorithm for hospitals to follow could have far‐reaching impact on making practice visible, auditing, and reporting practices enabling the achievement of best practice, as well as providing a consistent measure for future research.

## CONFLICTS OF INTEREST

The authors declare that they have no conflicts of interest.

## CONTRIBUTIONS

KB and KC substantially contributed to the creation of the algorithm. JS substantially contributed to the data and analysis, with KB and KC, of the Australian caesarean data into the algorithm. YT substantially contributed to the data and analysis, with KB and KC, of the Japanese vaginal birth data into the algorithm. All authors contributed to the writing of the paper. All authors contributed to the interpretation of the data, drafting and revising the article, and final approval of the version to be published.
